# Internet Search Trends for Common Hand Surgery Diagnoses

**DOI:** 10.7759/cureus.49755

**Published:** 2023-11-30

**Authors:** William Kilgallen, Brandon Earp, Dafang Zhang

**Affiliations:** 1 Orthopedics, Brigham and Women’s Hospital, Boston, USA; 2 Orthopedic Surgery, Brigham and Women’s Hospital, Boston, USA

**Keywords:** seasonal variation, online, internet, health literacy, hand surgery

## Abstract

Purpose: The internet is a common resource for patients seeking health information. Trends in internet search interests for common hand surgery diagnoses and their seasonal variations have not been previously studied. The objectives of this study were (1) to describe the temporal trends in internet search interest for common hand surgery diagnoses in the recent five-year time period and (2) to assess seasonal variations in search term interest.

Methods: An internet-based study of internet search term interest of 10 common hand surgery diagnoses was performed using Google Trends (Google, Inc., Mountain View, CA) from January 2017 to December 2021. The 10 diagnoses were “carpal tunnel syndrome,” “trigger finger,” “thumb arthritis,” “ganglion cyst,” “de Quervain’s tenosynovitis,” “lateral epicondylitis,” “Dupuytren disease,” “distal radius fracture,” “finger fracture,” and “scaphoid fracture.” Analysis of variance (ANOVA) was used to assess for seasonal differences in search interest, and temporal trends were assessed using the two-tailed Mann-Kendall trend test.

Results: During the study period, there was an increasing trend for search interest for “carpal tunnel syndrome,” “trigger finger,” “thumb arthritis,” “Dupuytren disease,” and “finger fracture,” both in the United States and worldwide. There was no significant temporal trend for “ganglion cyst,” “de Quervain’s tenosynovitis,” “lateral epicondylitis,” and “distal radius fracture.” There were no significant temporal trend for “scaphoid fracture” in the United States and a decreasing trend worldwide. There was significant seasonal variation in search term interest for “finger fracture” in the United States, “finger fracture” worldwide, and “scaphoid fracture” in the United States, with popularity peaking in the fall.

Conclusions: Despite growth in global internet usage, internet search interest has remained stagnant for many common hand surgery conditions, which may represent a shifting preference for patients to obtain health information from other resources. Internet search interest for traumatic hand conditions corresponds to seasonal variations in fracture epidemiology and peaks in the fall season.

## Introduction

The internet is a common resource for health information for hand surgery patients, both before and after consultation with a hand specialist. Nearly half of hand surgery patients research their condition on the internet prior to their initial consultation, and the utilization of online resources can be expected more in younger patients and patients with non-traumatic conditions [1]. Therefore, efforts have been made by professional societies to ensure that patients of all health literacy backgrounds have access to high-quality hand surgery educational information on the internet [1-6].

Approximately one-third of patients use the internet for self-diagnosis prior to initial consultation with a hand specialist [1]. Despite widespread utilization, the majority of patient self-diagnoses from internet searches do not match the ultimate diagnosis from the treating hand surgeon [7]. Yet, online health information has the capacity to frame patients’ understanding of their hand pathology and may affect subsequent discussions and decision-making in the surgeon’s office [1,7]. Often, additional counseling is necessary if patients present with discordant preconceived expectations [1].

The internet will continue to be an important resource for patients seeking information on their symptoms and/or diagnosis. The current state of internet utilization for common hand surgery conditions is relevant to practicing hand surgeons insofar as it influences patient expectations and in-office counseling. Trends in internet search interests for common hand surgery diagnoses and their seasonal variations have not been previously studied. The objectives of this study were (1) to describe the temporal trends in internet search interest for common hand surgery diagnoses in the recent five-year time period and (2) to assess seasonal variations in search term interest. We hypothesized that a significant increasing trend would exist in internet search interest for common hand conditions. We hypothesized that seasonal variations in search term interest would exist for traumatic conditions but not for non-traumatic conditions.

## Materials and methods

An internet-based study was performed without clinical subjects, and institutional review board approval was not required and not obtained. In July 2022, a study of the internet search term popularity of 10 common hand surgery diagnoses was performed using Google Trends (Google, Inc., Mountain View, CA) [8]. The 10 diagnoses were “carpal tunnel syndrome,” “trigger finger,” “thumb arthritis,” “ganglion cyst,” “de Quervain’s tenosynovitis,” “lateral epicondylitis,” “Dupuytren disease,” “distal radius fracture,” “finger fracture,” and “scaphoid fracture.” Internet search term interest was assessed both within the United States and worldwide during the five-year time period from January 2017 to December 2021. Google Trends provides the search interest over time as a number between zero and 100, where a value of 100 represents the peak popularity of the search term in the given region in the given time period, a value of 50 represents half popularity relative to the peak, and a value of zero represents insufficient data [8-10].

Monthly interest during the five-year study period for our search terms was collected. To assess for seasonal variations in search interest, dates were categorized by season. The seasons were classified as follows: spring (March, April, and May), summer (June, July, and August), fall (September, October, and November), and winter (December, January, and February). The analysis of variance (ANOVA) test was used to assess for differences in search interest by season. Furthermore, temporal trends from January 2017 to December 2021 for the 10 search terms, both in the United States and worldwide, were assessed using the two-tailed Mann-Kendall trend test. Kendall’s tau coefficient and its associated p-values were calculated. Kendall’s tau coefficient was classified as strong for 0.30 or above, moderate for 0.20-0.29, weak for 0.10-0.19, and very weak for below 0.10. The standard significance criterion of α < 0.05 was used, and temporal trends were assigned as increasing, no trend, or decreasing at the standard 95% level of confidence.

## Results

For each of the 10 common hand surgery diagnoses, the monthly internet search term interest over time was obtained from January 2017 to December 2021.

Through the five-year study period, we observed trends toward increased search term popularity for “carpal tunnel syndrome,” “trigger finger,” “thumb arthritis,” “Dupuytren disease,” and “finger fracture,” both in the United States and worldwide (p < 0.05). The magnitude of the increasing trends was strong for all of the above, except for “Dupuytren disease” worldwide. There was no significant temporal trend for the search terms “ganglion cyst,” “de Quervain’s tenosynovitis,” “lateral epicondylitis,” and “distal radius fracture.” There was no significant temporal trend in the search term “scaphoid fracture” in the United States but a moderate decreasing trend worldwide (p < 0.05) (Table 1).

**Table 1:** Temporal trends in the internet search interest of common hand surgeon diagnoses in the United States and worldwide. †Kendall’s tau and its associated p-values are calculated for the five-year timeframe from 2017 to 2021 to assess for monotonic temporal trends across the years of the study period. ‡Temporal trends are assigned at the 95% level of confidence.

Search term	Location	Kendall’s tau†	p-value	Temporal trend‡
Carpal tunnel syndrome	United States	0.351	<0.05	Increasing
	Worldwide	0.340	<0.05	Increasing
Trigger finger	United States	0.620	<0.05	Increasing
	Worldwide	0.640	<0.05	Increasing
Thumb arthritis	United States	0.445	<0.05	Increasing
	Worldwide	0.436	<0.05	Increasing
Ganglion cyst	United States	-0.069	0.4	No trend
	Worldwide	0.101	0.3	No trend
de Quervain’s tenosynovitis	United States	-0.041	0.6	No trend
	Worldwide	0.113	0.2	No trend
Lateral epicondylitis	United States	0.175	0.05	No trend
	Worldwide	0.144	0.1	No trend
Dupuytren disease	United States	0.305	<0.05	Increasing
	Worldwide	0.186	<0.05	Increasing
Distal radius fracture	United States	0.173	0.06	No trend
	Worldwide	0.080	0.4	No trend
Finger fracture	United States	0.309	<0.05	Increasing
	Worldwide	0.331	<0.05	Increasing
Scaphoid fracture	United States	-0.150	0.1	No trend
	Worldwide	-0.295	<0.05	Decreasing

**Figure 1:**
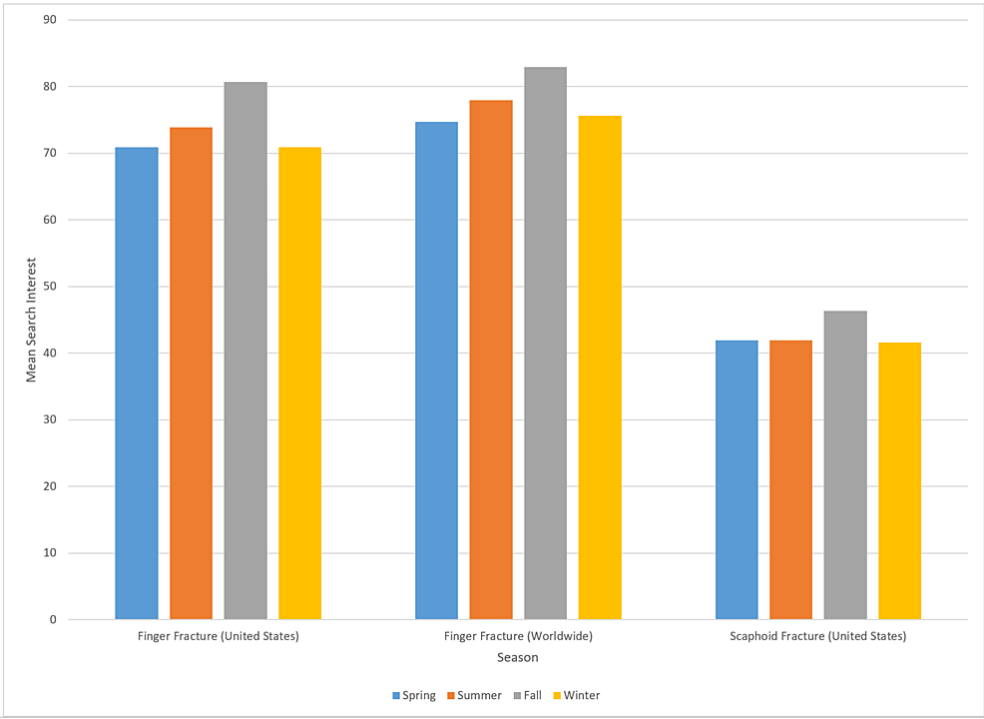
Bar graph depicting the search interest for the search terms with statistically significant seasonal variation.

We observed significant seasonal variation in search term interest for “finger fracture” in the United States (p < 0.05), “finger fracture” worldwide (p < 0.05), and “scaphoid fracture” in the United States (p < 0.05). In each of these cases, search term popularity peaked in the fall season. Seasonal variation in search term interest for “scaphoid fracture” worldwide did not reach statistical significance (p = 0.06). Search interest for the remaining search terms showed no statistically significant seasonal variation (Tables 2, 3 and Figure 1).

**Table 2:** Seasonal variation in the internet search interest (mean ± standard deviation) of common hand surgeon diagnoses in the United States.

Search term	Spring	Summer	Fall	Winter	p-value
Carpal tunnel syndrome	80.6 ± 9.5	82.2 ± 6.4	80.0 ± 6.2	77.4 ± 8.4	0.4
Trigger finger	86.3 ± 9.7	77.5 ± 12.8	81.6 ± 9.2	78.7 ± 10.5	0.1
Thumb arthritis	69.9 ± 10.4	70.6 ± 8.2	75.6 ± 7.8	76.9 ± 13.7	0.2
Ganglion cyst	85.1 ± 10.4	88.9 ± 5.6	86.8 ± 5.6	83.4 ± 6.7	0.2
de Quervain’s tenosynovitis	85.2 ± 10.0	86.5 ± 5.2	81.9 ± 5.4	82.7 ± 8.4	0.3
Lateral epicondylitis	74.5 ± 6.8	77.6 ± 3.7	74.5 ± 3.3	73.1 ± 6.4	0.1
Dupuytren disease	57.9 ± 11.9	61.4 ± 7.4	64.7 ± 14.7	61.7 ± 12.1	0.5
Distal radius fracture	70.5 ± 7.8	73.7 ± 3.6	72.1 ± 6.8	68.7 ± 6.9	0.2
Finger fracture	70.9 ± 10.6	73.9 ± 8.5	80.7 ± 9.9	70.9 ± 8.7	<0.05
Scaphoid fracture	41.9 ± 5.9	41.9 ± 2.4	46.3 ± 4.9	41.6 ± 4.5	<0.05

**Table 3:** Seasonal variation in the internet search interest (mean ± standard deviation) of common hand surgeon diagnoses worldwide.

Search term	Spring	Summer	Fall	Winter	p-value
Carpal tunnel syndrome	89.4 ± 7.1	88.1 ± 4.8	91.0 ± 3.4	88.3 ± 6.2	0.5
Trigger finger	69.8 ± 12.5	75.0 ± 9.6	71.1 ± 7.8	67.9 ± 7.7	0.2
Thumb arthritis	78.5 ± 11.0	75.9 ± 7.8	79.9 ± 7.8	81.0 ± 12.0	0.5
Ganglion cyst	81.7 ± 9.9	82.7 ± 4.7	83.4 ± 3.5	80.8 ± 8.2	0.8
de Quervain’s tenosynovitis	72.7 ± 9.7	70.1 ± 4.5	69.6 ± 2.8	69.2 ± 10.1	0.6
Lateral epicondylitis	76.7 ± 6.8	76.1 ± 3.2	77.3 ± 3.9	74.5 ± 7.4	0.5
Dupuytren disease	73.1 ± 12.5	71.5 ± 6.1	78.3 ± 9.3	77.6 ± 10.4	0.2
Distal radius fracture	80.5 ± 7.0	83.9 ± 3.2	82.9 ± 4.2	80.4 ± 5.3	0.2
Finger fracture	74.7 ± 9.6	77.9 ± 6.5	82.9 ± 7.9	75.6 ± 8.7	<0.05
Scaphoid fracture	55.3 ± 7.9	57.8 ± 3.3	59.7 ± 4.8	55.1 ± 4.1	0.06

## Discussion

The internet is a frequent resource of health information for hand surgery patients, and the utilization of the internet for self-diagnosis and/or supplemental information is likely to continue [1,2]. Trends in internet search volume for common hand surgery diagnoses across time, as well as seasonal variations in search volume, have not been previously studied. In this study, we have demonstrated an increasing temporal trend for internet search interest for some, but not all, hand surgery diagnoses, namely, for “carpal tunnel syndrome,” “trigger finger,” “thumb arthritis,” “Dupuytren disease,” and “finger fracture.” We have demonstrated seasonal variation in internet search interest for some traumatic hand surgery conditions, including “finger fracture” and “scaphoid fracture,” with the peak interest occurring in the fall.

In the last five years, internet search interest has increased for some common hand surgery diagnoses but not others. It is estimated that nearly five billion people worldwide, or nearly two-thirds of the world population, have access to the internet [11]. In the younger population between the ages of 15 and 24 years, approximately 71% of the world population have access to the internet [12]. In the last three years, it is estimated that nearly 800 million new users gained access to the internet [11,12]. Due to the continued growth of the internet, we hypothesized that increasing trends would exist in internet search interest for common hand conditions. This was in fact the case for the common hand diagnoses of “carpal tunnel syndrome,” “trigger finger,” “thumb arthritis,” “Dupuytren disease,” and “finger fracture,” both in the United States and worldwide. Moreover, Kendall’s tau coefficient supported a strong temporal trend in nearly all the above cases. However, despite the linear growth in global internet usage [11], internet search interest has remained stagnant for many common hand surgery conditions, namely, “ganglion cyst,” “de Quervain’s tenosynovitis,” “lateral epicondylitis,” and “distal radius fracture.” This may represent a shifting preference for patients to obtain health information from other resources. Patients may have good reason to be wary of health information on the internet, as the information on many websites has been shown to be of low quality [13,14]. In a survey study of 403 patients in an outpatient orthopedic fracture clinic by Jariwala et al., 70% of the respondents reported that health information they obtained on the internet differed from that obtained from the physician encounter [15]. In a survey study of 226 patients in an outpatient orthopedic hand clinic by Rao et al., 81% of the respondents reported that they preferred to receive health information by verbal communication, and 23% of the respondents reported that they would definitely not use the internet to learn about their diagnosis [1]. Alternative methods of engaging this subset of patients include in-office counseling, in-print or electronic educational handouts, or directed links to high-quality websites such as HandCare.org, the official online patient resource website of the American Society for Surgery of the Hand (ASSH).

Our hypothesis that seasonal variations in search term interest exist for traumatic conditions, but not for non-traumatic conditions, was supported by our findings. Internet search interest for the search terms “finger fracture” and “scaphoid fracture” varied by season, peaking in the fall. Multiple prior studies of fracture epidemiology have shown that fracture risk is highest in the summer months, likely due to lifestyle and activity [16-18]; however, when examining monthly fracture incidence, peak fracture risk often occurs in the month of September [17,18]. This would explain the peak in internet search interest for traumatic conditions in the fall, as patients seek online information after injury and in the convalescent period.

There are several limitations to this study. First, we have quantified internet search interest using Google Trends, which is only one of many potential search engines that patients may use. We focused on Google because it is the most commonly utilized search engine, accounting for approximately 85% of internet searches [19]. Second, with the data provided by Google Trends, we were able to comment on relative search interest over a specific time period but not the absolute search volume. Similarly, we were not able to put the search interest in hand conditions in the context of internet usage overall during the study time period. Third, search engines such as Google generally lead patients to a third-party website as the ultimate source of health information. This study was not able to identify the ultimate source of health information, which may have ranged from high-quality resources such as institutional or professional society websites to lower-quality resources such as sponsored content and social media websites [6,13,14]. Fourth, internet searches for common hand surgery diagnoses do not always represent patients seeking health information but may come from a variety of interested parties, such as healthcare providers and surgical trainees.

## Conclusions

The internet is an important resource of health information for many patients and has the capacity to instill preconceived expectations and frame subsequent in-office discussions. Usage trends and seasonal variations in internet searches for common hand surgery conditions are relevant to practicing hand surgeons because they influence patient expectations and counseling during consultations. In the last five years, as global internet usage has grown, internet search interest for some common hand surgery diagnoses has increased but not for others. This may represent an important shift in patient preferences to obtain health information from other resources. Alternatively, this may reflect changes in the incidence of some hand surgery conditions. There is no seasonal variation in internet search interest for non-traumatic hand conditions. Internet search interest for traumatic hand conditions corresponds to seasonal variations in fracture epidemiology and peaks in the fall season. Understanding the seasonal variation of internet search interest in traumatic hand conditions can help hand surgeons focus efforts on patient education, community outreach, and injury prevention. Seasonally timed injury prevention campaigns, such as via social media, may have a higher yield when disseminated in peak interest seasons.
